# Mitochondrial Dynamics in the Metabolic Memory of Diabetic Retinopathy

**DOI:** 10.1155/2022/3555889

**Published:** 2022-03-31

**Authors:** Ghulam Mohammad, Renu A. Kowluru

**Affiliations:** Department of Ophthalmology, Visual & Anatomical Sciences, Wayne State University, Detroit, MI, USA

## Abstract

Mitochondria play a central role in the development of diabetic retinopathy and in the metabolic memory associated with its continued progression. Mitochondria have a regulated fusion fission process, which is essential for their homeostasis. One of the major fission proteins, dynamin-related protein 1 (Drp1), is recruited to the mitochondria by fission protein 1 (Fis1) to initiate fragmentation. Our aim is to investigate the role of Drp1 in the altered mitochondrial dynamics in the continued progression of diabetic retinopathy. *Methods*. Drp1 activation, mitochondrial transport, and Drp1-Fis1 interactions were analyzed in retinal endothelial cells incubated in 20 mM glucose (HG), followed by 5 mM glucose (NG), for four days each (HG-NG group). The results were confirmed in retinal microvessels from streptozotocin-induced diabetic rats with poor glycemia (>350 mg/dl blood glucose, PC group), followed by normal glycemia (~100 mg/dl), for four months each (PC-GC group). *Results*. GTPase activity of Drp1, Fis1-Drp1 interactions, mitochondrial levels of Drp1, and fragmentation of the mitochondria were elevated in HG group. Mitochondrial Division Inhibitor 1 (Mdiv) or *Drp1*-siRNA attenuated Drp1 activation, mitochondrial fragmentation, and DNA damage. In HG-NG group, NG failed to ameliorate Drp1 activation and Drp1-Fis1 interactions, and the mitochondria remained fragmented. However, Mdiv supplementation in normal glucose, which had followed four days of high glucose (HG-NG/Mdiv group), inhibited Drp1 activation, mitochondrial fragmentation, and increase in ROS and prevented mitochondrial damage. Retinal microvessels from the rats in PC and PC-GC groups had similar Drp1 activation. *Conclusion*. Thus, Drp1 plays a major role in mitochondrial homeostasis in diabetic retinopathy and in the metabolic memory phenomenon associated with its continued progression. Supplementation of normal glycemia with a Drp1 inhibitor could retard development and further progression of diabetic retinopathy.

## 1. Introduction

Diabetic retinopathy is among the leading causes of vision loss, but the multifactorial nature of this blinding disease has limited therapeutic options for its treatment. Circulating high glucose damages the retinal structure, function, and its vasculature and initiates many metabolic abnormalities; retinal mitochondrial structure, function, and DNA are damaged in diabetes, and cytochrome C leaks out in the cytosol, accelerating apoptosis of capillary cells. This leads to the degeneration of retinal capillaries, and poor or no perfusion of these acellular capillaries subsequently results in neovascularization [1–5].

Mitochondria are highly dynamic structures, and these dynamic properties are critical for their optimal function in energy generation, as mitochondrial size, shape and network are controlled by cell physiology. They continuously undergo fission and fusion, and the interplay of fusion and fission helps mitochondria in efficient oxidative phosphorylation [6]. In addition to structural and DNA damage in diabetic retinopathy, mitochondria are also fragmented [7]. Mitochondria have double membranes, and their fusion needs fusion of both outer and inner membranes; while optic atrophy mediates inner membrane fusion, mitofusins 1 and 2 (Mfn1 and Mfn2) help in outer membrane fusion, and among the two mitofusins, Mfn2 is considered essential for fusion [8]. In diabetic retinopathy, Mfn2 is downregulated, and its overexpression prevents glucose-induced increase in mitochondrial fragmentation and preserves their functional and genomic stability [7]. Several proteins are implicated in the mitochondrial fission process, and among those, a GTPase Dynamin-related protein 1 (Drp1) is considered as the major fission protein [9]. Drp1 is a cytosolic protein, and its recruitment inside mitochondria is mediated by proteins such as fission protein 1 (Fis1), mitochondrial fission factor and mitochondrial elongation factors 1 and 2. Among this group of proteins, Fis1 is considered as the most critical protein required for mitochondrial fragmentation in mammalian cells [10, 11]. Drp1 is localized to the outer membrane of the mitochondria via its C-terminal, and it is recruited to the mitochondrial surface to bind to Fis1; interactions of Drp1 with Fis1 regulate GTPase activity of Drp1 [12, 13]. The role of Drp1-Fis1 in mitochondrial homeostasis in diabetic retinopathy is not clear.

Epidemiology of diabetes interventions and complications studies that followed the Diabetes Control and Complications Trial have shown that the clinical features of retinopathy continue to develop long after hyperglycemia is reversed by normal glycemia, suggesting a “metabolic memory” phenomenon [14–17]. This metabolic memory phenomenon can be duplicated in both *in vitro* and *in vivo* experimental models of diabetic retinopathy; apoptosis of capillary cells and histopathology characteristic of diabetic retinopathy initiated during prior poor glycemic control in rodents and dogs fail to show any benefit from the intensive glycemic control, which follows it. Similarly, apoptosis continues in retinal capillary cells even when they are incubated in normal glucose, which has followed high-glucose exposure [18–20]. We have shown that mitochondria remain dysfunctional with damaged structures and genome, and the compromised electron transport chain continues to fuel the self-perpetuating vicious cycle of free radicals [21–23]. In addition, due to sustained hypermethylation of *Mfn2* promoter, it continues to be suboptimal, and mitochondria remain fragmented [23]. However, the role of Drp1 in maintaining mitochondrial dynamics in the continued progression of diabetic retinopathy remains elusive.

This study is aimed at investigating the role of Drp1 in the compromised mitochondrial dynamics in the progression of diabetic retinopathy. Using human retinal endothelial cells (HRECs) in culture and the rat model of diabetic retinopathy, we have investigated the effect of hyperglycemia on Drp1-Fis1 interactions and mitochondrial transport of Drp1, and the results are confirmed in retinal microvessels from human donors with diabetic retinopathy. We have also examined the effect of intervention of good glycemic phase with Drp1 inhibitors on the mitochondrial dynamics.

## 2. Methods

### 2.1. Retinal Endothelial Cells

Endothelial cells isolated from nondiabetic human retina (HRECs Cell system, Kirkland, WA) from 6 to 8^th^ passage were incubated in 5 mM D-glucose (NG) or 20 mM D-glucose (HG) in DMEM containing 1% fetal calf serum, 9% Nu-serum, and 1 *μ*g/ml endothelial growth supplement for four days [7, 24]. High-glucose exposed cells were divided into two groups, cells in group 1 remained in continuous high glucose for eight days, in the absence or presence of 100 *μ*M, mitochondrial division inhibitor 1, Mdiv (HG and HG/Mdiv groups, respectively; Cat no. 475856; Sigma-Aldrich Corp., St. Louis, MO) [25]. In group 2, after four days of 20 mM D-glucose exposure, cells were incubated in 5 mM D-glucose for four additional days in a medium supplemented with or without Mdiv (HG-NG/mdiv and HG-NG, respectively). The duration of high-glucose exposure is based on our previous reports showing that mitochondrial damage and decrease in mitochondrial copy numbers are not seen till the duration is extended beyond 48 hours [26–28]. For *Drp1*-siRNA experiments, a group of cells in 5th-7th passage were transfected with *Drp1-* siRNA (Cat no. AM16708, siRNA ID: 138220, Thermofisher Scientific, Waltham, MA, USA) using Lipofectamine™ RNAiMAX Transfection Reagent (Cat. No. 13778150, Invitrogen™, Carlsbad, CA,USA) employing the methods described previously [22, 26–28]. Parallel transfection was performed using nontargeting scrambled control RNA (Cat no. AM4611, Thermofisher Scientific). After transfection, the cells were incubated in 5 mM or 20 mM D-glucose media for four days. Please note that due to metabolic alterations induced by prior high-glucose exposure in HRECs, *Drp1*-siRNA was not used in the reversal experiments. As an osmotic/metabolic control, each experiment had a parallel incubation where HRECs were incubated in 20 mM L-glucose (L-Gl), instead of 20 mM D-glucose. Experiments were run using HRECs from the same passage and from the same batch, and these incubation conditions do not affect the cell phenotype and are routinely used in our laboratory [22, 23].

### 2.2. Rats

Diabetes was induced in male Wistar rats (~200 g body weight) by intraperitoneal streptozotocin administration (55 mg/kg BW), and diabetic rats were divided into three groups. Group 1 rats remained in poor glycemic control (PC, blood glucose > 350 mg/dl) throughout the eight-month duration. Group 2 rats were in poor glycemic control for four months followed by good glycemic control (blood glucose less than 150 mg/dl) for another four months (PC-GC), and rats in group 3 were maintained in good glycemic control soon after induction of diabetes for the entire eight months (GC). Good glycemic control was maintained by administering insulin two times a day (total of 5-7 IU/day) [22, 29]. Our choice of duration of diabetes was based on our previous reports showing that mitochondrial damage and decrease in mtDNA copy numbers are not seen in the retina at two months of diabetes in rats, but are significant at 6-8 months of diabetes [26–28]. The experimental protocols were approved by Wayne State University's Animal Care and Use Committee, and the treatment of the animals followed the guidelines of the Association for Research in Vision and Ophthalmology Resolution with the Use of Animals in Research.

### 2.3. Human Subjects

Eye globes, enucleated within 6-9 hours of death, from donors 55 to 75 years of age and from their age- and sex-matched nondiabetic donors (Table 1), were obtained from the Eversight Eye Bank (Ann Arbor, MI). Each diabetic donor had documented retinopathy (nonproliferative or proliferative). The eye globes received from the Eye Bank were coded and had no patient ID.

### 2.4. Retinal Microvessels

Microvessels were prepared by incubating the rat retina (1/2 retina) or human retina (1/8^th^ retina) in 10-15 ml distilled water for one hour at 37°C. This was followed by gentle removal of the nonvascular tissue under a microscope, as described previously [7, 24].

### 2.5. Gene Expression

RNA (extracted by Trizol) was used to prepare cDNA using a High-Capacity cDNA Reverse Transcription kit (Applied Biosystems, Foster City, CA). Employing gene- and species-specific primers (Table 2), SYBR green-based real-time quantitative PCR (qRT-PCR) was performed. *β*-Actin (Human-*ACTB* and Rat-*Actb*) was employed as a housekeeping gene, and relative gene expression was calculated by the Delta-Delta-Ct method [7, 22].

### 2.6. GTPase Activity of Drp1

Drp1 activity was measured using the GTPase assay kit (DATG-200; Bioassay Systems, Hayward, CA), following the manufacturer's instructions, as reported by others [30] and recently used by us [31]. Samples (HRECs or microvessels) were lysed in lysis buffer (50 nM HEPES, pH 7.5, 120 mM NaCl, 5 mM EDTA,10 mM sodium pyrophosphate, 50 mM NaF, 1 mM Na3VO4, 1% Triton X-100, and protease inhibitors), and 200 *μ*g protein was immunoprecipitated using 5 *μ*g Drp1 monoclonal antibody (Cat. No. sc-101270, Santa Cruz Biotechnology, Santa Cruz, CA). The pellet was incubated with Protein A/G Plus agarose beads suspended in lysis buffer. After washing with Tris buffer (50 mM Tris, pH 7.5, 2.5 mM MgCl2, and 0.02% *β*-mercaptoethanol), the beads were incubated with 0.5 mM GTP at room temperature for 30 minutes. The phosphate released by the conversion of GTP into GDP was quantified at 620 nm.

### 2.7. Immunofluorescence Microscopy

Mitochondrial localization of Drp1 was performed in HRECs by immunofluorescence technique using the mitochondrial marker Tom20 (Cat. No. MABT166, EMD Millipore, Temecula, CA; 1 : 200 dilution) and Drp1 antibodies (Cat. No ab184247, Abcam, Cambridge, MA; 1 : 200 dilution). Secondary antibodies included Alexa Fluor-488 (green), conjugated anti-rabbit (Cat. No. A11008, Molecular Probes-Life Technologies, Grand Island, NE; 1 : 500 dilution), and Texas red-conjugated anti-mouse (Cat. No. TI200, Vector Laboratories, Burlingame, CA; 1 : 500 dilution). Immunolabelled cells were mounted using DAPI-containing (blue) Vectashield mounting medium (Vector Laboratories) to counterstain the nuclei. ZEISS proinbuilt software package and modules were used to calibrate the captured images, and Pearson's correlation was calculated using a random region of interest from 25 to 30 cells in each group by employing the colocalization software module [31, 32].

Colocalization of Drp1, Fis1 and Tom20 was performed using Drp1 and Tom20 primary antibodies and DyLight® 350 (Cat. no. ab201797, Abcam)-conjugated Fis1 mouse primary antibody (Cat. No. MA5-27834; Thermo Scientific), and each antibody was used at 1 : 100 dilution. Cells were imaged under a Zeiss Apotome (Carles Zeiss, Inc., Chicago, IL, USA) [7, 24] at 40x magnification. Zeiss proinbuilt software package and modules were employed to calibrate the images.

### 2.8. Mitochondrial Fission

Live cell microscopy was performed to evaluate mitochondrial fission, as reported previously. Coverslips were incubated with 200 nM MitoTracker green FM (Cat. No. M7514, Thermo Fisher Scientific) for 30 minutes, followed by rinsing the coverslips with PBS and imaging them using Zeiss Apotome microscope at 63x magnification [7, 31]. Six to eight cells in each group were imaged/experimented, and each experiment was repeated in three different cell preparations.

### 2.9. Mitochondrial Damage

Mitochondrial damage was evaluated by quantifying the levels of reactive oxygen species (ROS) and mitochondrial membrane potential and DNA (mtDNA) damage.

ROS levels were quantified by incubating HRECs with a mitochondrial superoxide indicator, MitoSOX red, 5 *μ*M (Cat. No. M36008, Thermo Fisher Scientific), in the presence of 200 nM MitoTracker green for 30 minutes at 37°C. Six to eight cells/group were imaged under a Zeiss Apotome at 63x magnification, and this was repeated in 3-4 different cell preparations. The intensity of the MitoSox was quantified by ImageJ software (ImageJ, U.S. National Institutes of Health, Bethesda, MD) [33].

Mitochondrial transmembrane potential was determined by using a mitochondrial binding dye, JC-1 (Cat. No. MP03168, Molecular Probes, Carlsbad, CA, USA), as described previously [33]. Briefly, after incubation, the cells were washed with PBS and incubated in DMEM containing 5 *μ*M JC-1 for 30 minutes at 37°C. The cells were then rinsed with PBS, and visualized under Zeiss Apotome fluorescence microscope at 20x magnification. The measurements were carried out at excitation wavelength of 485 nm and emission wavelength of 530 nm for monomers (green) or 525 nm and 590 nm for aggregates (red). Zeiss software module was used to calculate the ratio of red and green fluorescence [33].

Mitochondrial damage was also confirmed by quantifying the mRNA levels of mtDNA-encoded *cytochrome b* (*Cytb*) of complex III of the electron transport chain by qRT-PCR; *ACTB* was used as the housekeeping gene [33].

### 2.10. Statistical Analysis

The statistical analysis was performed using Graph Pad Prism (San Diego, CA), and the data are presented as mean ± SD. Group comparisons were performed using one-way ANOVA followed by Dunn's *t*-test; *p* < 0.05 was considered significant.

## 3. Results

### 3.1. *In Vitro* Model

#### 3.1.1. Effect of Reversal of High-Glucose Insult with Normal Glucose on Mitochondrial Integrity

**Figure 1:**
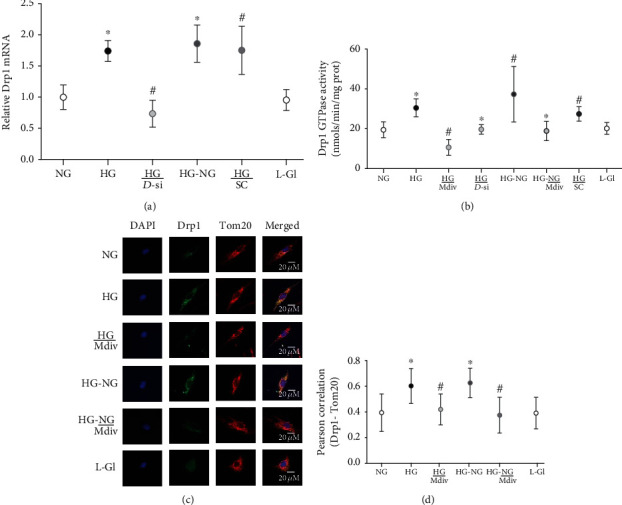
Drp1 activation and its continued mitochondrial localization remain high even after reversal of high-glucose insult. Drp1 (a) mRNA, (b) GTPase activity, and (c) its mitochondrial localization of Drp1. Each image in (c) is a representative of 5-7 cells/group, repeated in 3 or more cell preparations, with Drp1 is in green and Tom20 in red. (d) Pearson correlation coefficient between Drp1-Tom20 was calculated from 25 to 30 cells in each group. The values in the graphs are represented as mean ± SD, obtained from 3 to 4 different cell preparations, with each measurement made in duplicate. NG and HG: HRECs in 5 mM or continuous 20 mM D-glucose, respectively; HG/Mdiv: HRECs in continuous 20 mM glucose with Mdiv; HG/*D*-si and HG/SC: *Drp1*-siRNA or control scrambled RNA transfected HRECs in continuous 20 mM glucose; HG-NG and HG-NG/Mdiv: HRECs in 20 mM D-glucose for four days followed by 5 mM glucose, without or with Mdiv, respectively, for four additional days; L-Gl: 20 mM L-glucose. ^∗^*p* < 0.05 vs. NG, ^#^*p* < 0.05 vs. HG.

Mitochondria are damaged in diabetic retinopathy and reinstitution of good glycemic control, after a period of poor glycemic control, fails to reverse the progression of diabetic retinopathy, and the dysfunctional mitochondria continue to produce excessive amounts of free radicals [3, 20]. Drp1, a cytosolic protein, in response to various cellular stimuli to regulate mitochondrial morphology, translocates to the mitochondria and promotes its fission [10]. mRNA of Drp1 and its GTPase activity were significantly higher in retinal endothelial cells incubated with high glucose, compared to cells in normal glucose (Figures 1(a) and 1(b)). As with total Drp1, high glucose also increased its mitochondrial expression as seen by increased colocalization of Drp1 with an outer mitochondrial membrane marker Tom20; Pearson correlation coefficient between Drp1-Tom20 in high glucose was significantly higher compared to cells in normal glucose (Figures 1(c) and 1(d)). Incubation of cells in 20 mM L-glucose had no effect on Drp1 mRNA and activity, and its mitochondrial localization (Figures 1(a)–1(d)).

**Figure 2:**
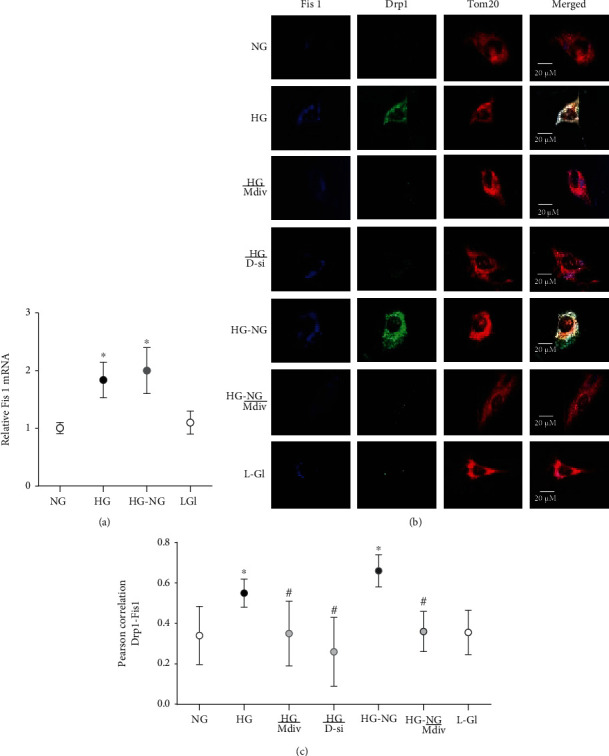
Interaction of Drp1 with Fis1 remains elevated even when high glucose is replaced by normal guclose. Fis1 (a) mRNA using *ACTB* as a housekeeping gene, and (b) its interaction with Drp1 by imaging cells at 40x magnification. (c) Represents Pearson correlation coefficient between Drp1 and Fis1 from 20 to 30 cells/group. Each measurement was made in 3-4 cell preparations. ^∗^*p* < 0.05 vs. NG, ^#^*p* < 0.05 vs. HG.

Drp1 is recruited to the mitochondrial surface where it binds to the receptor protein Fis1 [13, 34]; compared to cells in normal glucose, high glucose increased Fis1 expression (Figure 2(a)). Immunofluorescence staining showed increased costaining of Drp1-Fis-Tom20 in the mitochondria and higher Pearson correlation between Drp1-Fis1 (Figures 2(b) and 2(c)). Reversal of high-glucose insult to normal glucose (HG-NG group) failed to reverse Fis1 expression and its interaction with Drp1 (Figures 2(a)–2(c)). Values in HG and HG-NG groups were significantly higher than cells in normal glucose (*p* > 0.05).

**Figure 3:**
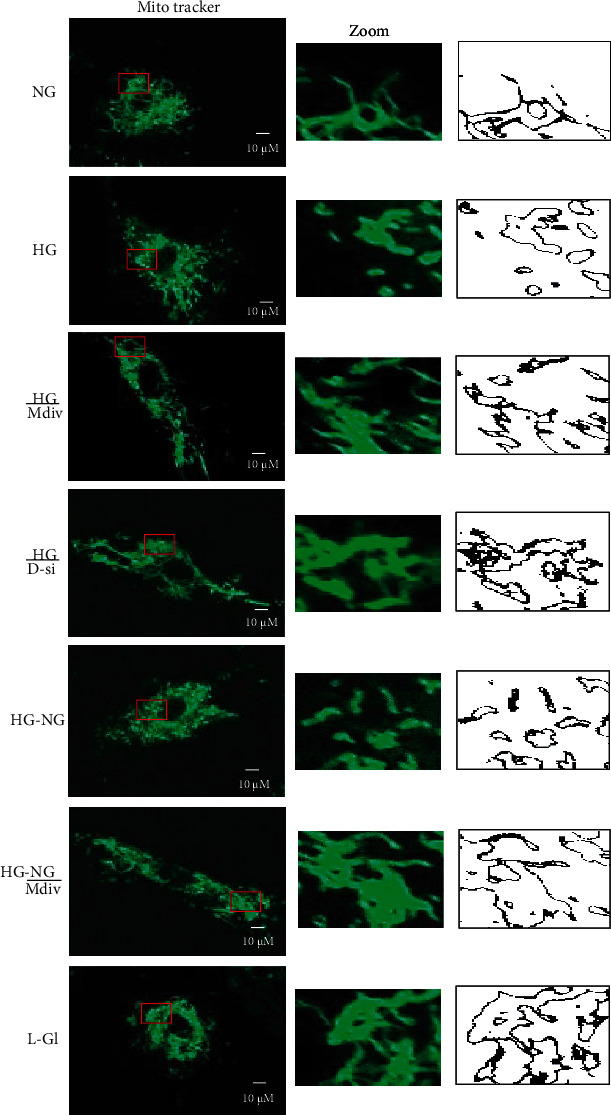
Direct inhibition of Drp1 during reversal phase prevents mitochondrial fragmentation. Mitochondrial morphology was evaluated by live cell microscopy (63x) using MitoTracker Green dye. The middle panel displays the area inside the box, and the right panel shows the analysis of the area inside the box by ImageJ for particles (size = 0‐infinity; circularity = 0 to 1) after adjusting for color threshold. Bare outlines were drawn to illustrate the morphology. Each image is representative of 5-7 cells/group, repeated in 2-3 different cell preparations.

Consistent with the failure to prevent activation of Drp1 and its mitochondrial accumulation, four days of normal glucose, after four days of high glucose (HG-NG), had no effect on mitochondrial fission in HRECs. Live cell imaging showed higher mitochondrial fragmentation in HG and HG-NG groups, compared to cells in normal glucose. Incubation of cells in 20 mM L-glucose had no effect on mitochondrial fragmentation (Figure 3).

**Figure 4:**
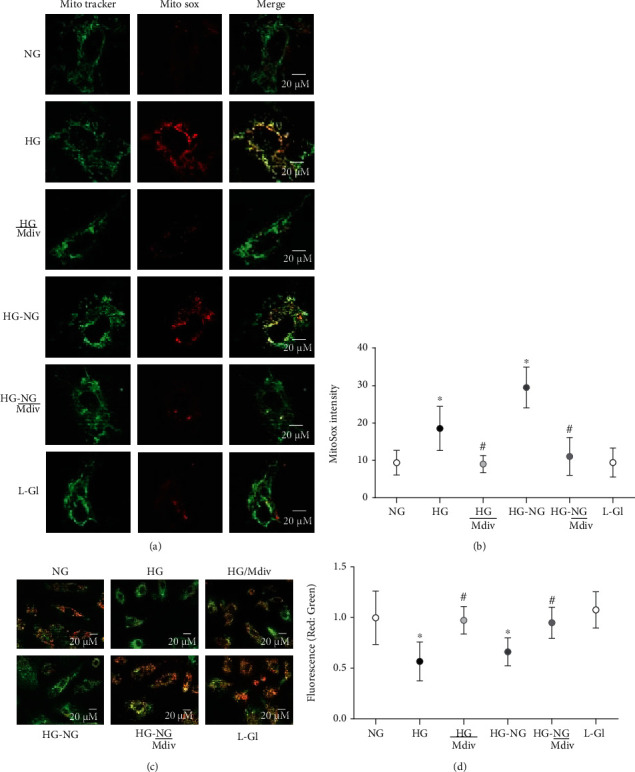
Amelioration of glucose-induced increase in mitochondrial ROS and membrane depolarization by Drp1 inhibition. (a) Cells stained with MitoSOX Red for mitochondrial ROS. (b) MitoSox fluorescence intensity, quantified using ImageJ, from 4 to 6 images in each group. (c) JC-1 staining of HRECs, green fluorescence represents the depolarized (monomer), and reddish-orange represents hyperpolarized (J aggregates) mitochondria. (d) Ratio of red and green fluorescence intensity in JC-1 stained cells. Each image is representative of more than 5-8 cells/group, and imaging was repeated in three different cell preparations. Mitosox intensity and the ratio of red to green fluorescence intensity are represented as mean ± SD. HG/Mdiv: HRECs in continuous 20 mM glucose in the presence of Mdiv; HG/*D*-si and HG/SC: *Drp1*-siRNA or control scrambled RNA transfected HRECs in continuous 20 mM glucose; HG-NG and HG-NG/Mdiv: HRECs in 20 mM D-glucose for four days followed by 5 mM glucose, without or with Mdiv, respectively, for four additional days; L-Gl: 20 mM L-glucose. ^∗^*p* < 0.05 vs. NG, ^#^*p* < 0.05 vs. HG.

**Figure 5:**
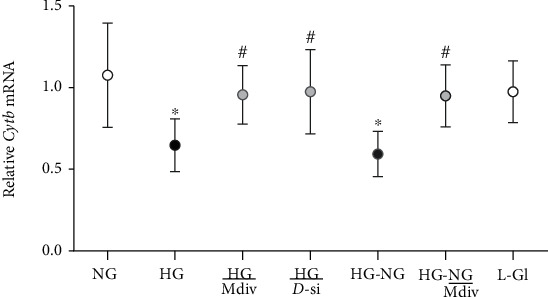
Direct inhibition of Drp1 prevents mtDNA damage. *Cytb* mRNA was quantified by qRT-PCR. HG/Mdiv: HRECs in continuous 20 mM glucose with Mdiv; HG/D-si and HG/SC = *Drp1*-siRNA or control scrambled RNA transfected HRECs in continuous 20 mM D-glucose; NG-HG and NG-HG/Mdiv: HRECs in 20 mM D-glucose for four days followed by 5 mM glucose, without or with Mdiv, respectively, for four additional days; L-Gl: 20 mM L-glucose. ^∗^*p* < 0.05 vs. NG, ^#^*p* < 0.05 vs. HG.

Accordingly, mitochondrial ROS and decrease in the mitochondrial transmembrane potential also continued to be elevated with reduced *Cytb* mRNA in HG-NG group compared to NG group (Figures 4 and 5). MitoSox intensity was significantly higher in HG and HG-NG groups, compared to NG group. Cells in 20 mM L-glucose had similar mitochondrial ROS content and membrane potential as obtained from cells in 5 mM D-glucose.

#### 3.1.2. Effect of Mitochondrial Fission Inhibitor or *Drp1*-siRNA on Glucose-Induced Drp1 Activation and Mitochondrial Integrity

**Figure 6:**
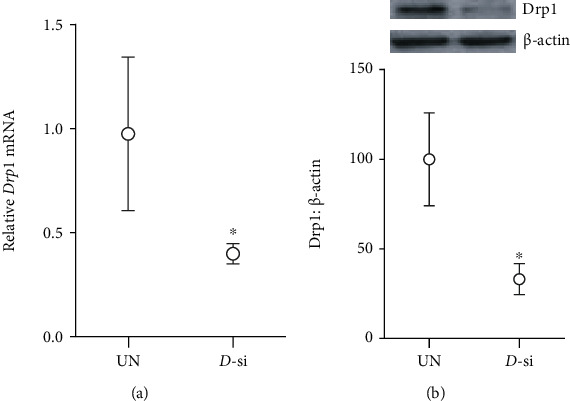
Drp1-siRNA transfection efficiency: cells transfected with Drp1-siRNA were analyzed for Drp1 (a) mRNA levels and (b) protein expression. Values are mean ± SD of two different transfections, each measurement made in triplicate. UN: untransfected cells; *D*-si: *Drp1*-siRNA transfected cells. ^∗^*p* < 0.05 vs. untransfected cells.

Mitochondrial fission inhibitor, Mdiv or Drp1-siRNA, prevented glucose-induced increase in Drp1 activity; the activity was significantly lower compared with the values obtained from cells in HG group (*p* < 0.05). Mdiv also prevented mitochondrial translocation of Drp1, and the Pearson correlation coefficients between Drp1 and Tom20 in HG/Mdiv and NG groups were not different from each other (*p* > 0.05), but were significantly different from HG group (*p* < 0.05; Figure 1). Inhibition of Drp1 also prevented the increase in Drp1-Fis1 interactions, mitochondrial fission, ROS levels, and its membrane potential; HG and HG/Mdiv groups had significantly different values (*p* < 0.05; Figures 2–4). Consistent with these, Mdiv or *Drp1*-siRNA also ameliorated glucose-induced decrease in *Cytb* transcription; compared to untransfected or scrambled RNA, transfected cells in high glucose mRNA of *Cytb* was significantly higher in Mdiv and *Drp1*-siRNA cells in high glucose (*p* > 0.05, Figure 5).  Figure 6 shows that the transfection efficiency of *Drp1-*siRNA in HRECs, as determined by its protein (Western blot) and mRNA, was ~60%.

#### 3.1.3. Effect of Direct Inhibition of Drp1 during the Reversal Phase of Mitochondrial Fission

Inclusion of Mdiv during the normal glucose phase, which followed four days of high-glucose exposure (HG-NG/Mdiv group), prevented the increase in Drp1 activity and its mitochondrial accumulation, which was induced by prior high glucose insult (Figures 1(b) and 1(c)). Mdiv supplementation during this normal glucose phase also prevented Drp1-Fis1 interactions and mitochondrial fission, and the values obtained from cells in HG-NG and HG-NG/Mdiv were different from each other (*p* < 0.05), but were similar to those obtained from cells in NG or HG/Mdiv groups (Figures 2(b), 2(c), and 3). In the same cell preparations, Mdiv addition during incubation in normal glucose, which had followed four days of high glucose, also prevented increase in mitochondrial ROS levels and its membrane potential, and decrease in *Cytb* mRNA; NG, HG/Mdiv, and HG-NG/Mdiv groups had similar values (Figures 4 and 5). Thus, “in principle,” compared to just normal glucose alone, direct inhibition of Drp1 during the reversal phase provided a beneficial effects on Drp1 activation and mitochondrial fission.

### 3.2. *In Vivo* Model

The role of mitochondrial fragmentation in the continued progression of diabetic retinopathy after termination of hyperglycemia was confirmed in a rat model. Rats in the PC group (continuous poor glycemic control for eight months) had significantly lower body weight (~350 g) and high blood glucose (>350 mg/dl) and urine output (>90 ml/24 hours), compared to the rats in Norm group (~550 g,~100 mg/dl, and 10-15 ml/24 hours, respectively). Rats in PC-GC group (four months of poor control, followed by four months of good glycemic control for four additional months) during their poor glycemic control had similar values for their body weight and glucose as in PC group. After four months of good glycemia in these rats, the values became similar to those in GC or norm group, and rats in GC and norm groups had similar body weights and blood glucose values.

**Figure 7:**
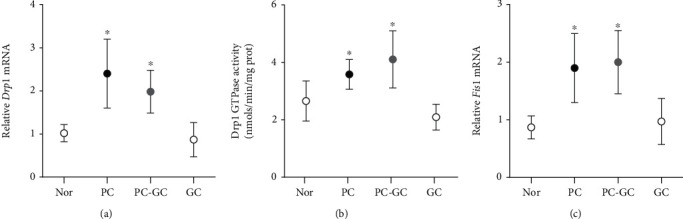
Effect of reinstitution of normal glycemia in rats after a period of hyperglycemia, on Drp1 and its receptors. Retinal microvessels were analyzed for (a) *Drp1* mRNA, (b) GTPase activity, and (c) *Fis1* mRNA. Each measurement was made in duplicate in 5-7 rats/group, and the values are represented as mean ± SD, Norm: normal; PC: rats in poor control for eight months; PC-GC: rats in poor control for four months followed by four months of good control; GC: rats in good glycemic control for eight months. ^∗^*p* < 0.05 vs. normal, ^#^*p* < 0.05 vs. PC.

In accordance with the results from endothelial cells in high glucose, reinstitution of good glycemic control after a period of poor glycemic control in rats failed to ameliorate the hyperglycemia-induced increase in *Drp1* mRNA in the retinal vasculature, and the GTPase activity of Drp1 also remained high; values obtained from rats in PC and PC-GC groups were not different from each other (*p* > 0.05), but were significantly higher than those in norm group (*p* < 0.05; Figures 7(a) and 7(b)). Similarly, Drp1 receptor protein *Fis1* also remained elevated even when the hyperglycemia was replaced by normal glycemia (Figure 7(c)). However, rats in continuous good control (GC group) had *Drp1* and *Fis1* gene expression and GTPase activity of Drp1 similar to that obtained from rats in norm group (*p* > 0.05), and these values were significantly different from the rats in PC-GC group (Figure 7).

### 3.3. Human Retinal Microvessels

**Figure 8:**
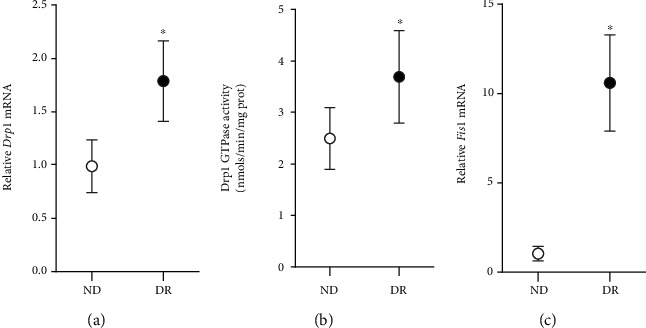
Drp1 and Fis1 in human donors with diabetic retinopathy. Retinal microvessels from donors with documented diabetic retinopathy (DR), and their age-matched nondiabetic donors (ND) were analyzed for (a) mRNA of *Drp1* (b) GTPase activity of Drp1, and (c) mRNA of *Fis1*. Each measurement was made in duplicate in 5-6 donors/group, and the values are represented as mean ± SD, ^∗^*p* < 0.05 vs. ND.

Transitioning to human disease, microvessels from human donors with diabetic retinopathy had higher gene transcript and GTPase activity of Drp1 compared to microvessels from age-matched nondiabetic human donors. In the same diabetic retinopathy donors, *Fis1* expression was also significantly higher (Figure 8).

## 4. Discussion

Mitochondria play a pivotal role in the development of diabetic retinopathy, and their sustained dysfunction is implicated in the metabolic memory associated with the continued progression of diabetic retinopathy even after termination of hyperglycemic insult [20, 23, 29, 35, 36]. Mitochondria are highly dynamic entities and continuously go through rapid and opposing processes of fission and fusion [37, 38]. The balance between these two opposite processes is important in regulating mitochondrial number and size, and any abnormality in either the fission or fusion machinery or both fusion and fission machineries, can result in impaired mitochondrial homeostasis [39]. Mitochondrial fusion requires Mfn2 and Opa1, and mitochondrial fission is mediated by the recruitment of cytosolic Drp1 to outer mitochondrial membrane proteins like Fis1 [13, 34]. Impaired mitochondrial dynamics is associated with many diseases including optic neuropathies [40]. Our previous work has shown that Mfn2 is decreased in the retinal vasculature in the hyperglycemic milieu, and reinstitution of good glycemia has no beneficial effect on its expression [23]. Furthermore, Drp1 and its GTPase activity are increased in hyperglycemia [31], and here, we show that Fis1 interaction with Drp1 and mitochondrial localization of Drp1 are also elevated. Reversal of hyperglycemia by normal glycemia does not ameliorate GTPase activity of Drp1 and its interaction with Fis1, and increased Drp1 accumulation in the mitochondria continues to fragment them. Consistent results from both *in vitro* (retinal endothelial cells exposed to high glucose, followed by normal glucose) and *in vivo* (retinal microvessels from diabetic rodents maintained in poor and good glycemic control) models demonstrate the role of Drp1-mitochondrial fragmentation in the development of diabetic retinopathy and in the metabolic memory associated with its continued progression. Our *in vitro* results show that direct targeting Drp1 activation during the reversal phase prevents continued activation of Drp1 and mitochondrial fragmentation. Overall, these results suggest that sustained activation of Drp1 during the normal glycemic phase, which has followed the hyperglycemic phase, does not allow mitochondrial fragmentation to halt, but the combination of good glycemia with a therapy targeting mitochondrial fission has the potential to protect them from fragmenting and prevent their dysfunction.

Drp1 is primarily located in the cytosol, but in response to various cellular stimuli to regulate mitochondrial morphology, it is translocated to the mitochondrial surface and assembles at endoplasmic reticulum-mitochondrial contact sites, inducing mitochondrial fission via its GTPase activity [10, 13, 41] Translocation of Drp1 in the mitochondria is increased significantly in high-glucose condition, and its GTPase activity is also increased [31]. Mitochondrial translocation of Drp1 and its GTPase activity are closely dependent on its interaction with Fis1 [13, 42], and our results show that glucose increases the interactions of Drp1 with Fis1, and mitochondrial fragmentation.

Mitochondrial division inhibitor 1, a quinazonilone derivative, is a selective cell-permeable inhibitor of Drp1 and inhibits Drp1-dependent mitochondrial fission [43]. It inhibits GTPase activity of Drp1 by blocking the self-assembly of Drp1, resulting in the irreversible formation of elongated and tubular mitochondria [44]. Here, we show that the addition of Mdiv inhibits glucose-induced increase in GTPase activity of Drp1, and this is further confirmed by using cells transfected with *Drp1*-siRNA. Mitochondria fusion-fission is essential to maintain mitochondrial morphology and metabolism and their quality control by autophagic clearance of the dysfunctional mitochondria [45]. Inhibition of Drp1-Fis1 interactions reduces pathological mitochondrial fission and rescue respiration [46, 47], and our results show that Mdiv or *Drp1*-siRNA inhibit glucose-induced mitochondrial fragmentation in endothelial cells. Morphology of the mitochondria is also important in maintaining mitochondrial function including ROS levels, ATP production, and apoptosis [48], and an increase in Drp1 and Drp1-Fis1 interactions are shown to increase ROS levels in many neurodegenerative diseases [49]. Thus regulation of Drp1 activation prevents glucose-induced mitochondrial damage and an increase in mitochondrial ROS.

Retinal microvessels, the site of histopathology characteristic of diabetic retinopathy, from diabetic rats and from human donors with documented diabetic retinopathy, also showed a significant increase in the mRNA levels of *Drp1* and *Fis1* and GTPase activity of Drp1, compared to their respective controls (age-matched normal rats and nondiabetic donors). Consistent results from human disease samples and *in vitro* and *in vivo* models strongly suggest the importance of Drp1 in diabetic retinopathy. In support, our previous work has shown that the mitochondrial fusion protein Mfn2 is also downregulated in retinal microvessels [7, 23], further confirming the role of mitochondrial homeostasis in diabetic retinopathy. In addition, mice with *Drp1* gene downregulated are protected from diabetes-induced increase in capillary cell apoptosis and degeneration and formation of pericyte ghosts [50].

As stated above, reversal of high glucose to normal glucose does not provide any relief to the dysfunction of mitochondria, and retinopathy continues to progress [20, 23, 29, 35, 36]. Our results show that incubation of HRECs in normal glucose for four days, which follows four days of high-glucose exposure, fails to reverse the increase in Drp1 and its GTPase activity, and mitochondrial levels of Drp1 and Drp1-Fis1 interactions remain elevated. Consistent with this, mitochondria remain fragmented with elevated ROS levels. In the same model, Mfn2 remains suboptimal [7, 23], suggesting that the mitochondria are subjected to double whammy with downregulated fusion machinery (Mfn2) and upregulated fission process (Drp1). Addition of Mdiv during those four days of normal glucose prevents the increase in GTPase activity of Drp1 and its mitochondrial levels and interactions with Fis1. Mitochondria become less fragmented, compared to cells with Mdiv, suggesting that in addition to normal glucose, direct inhibition of Drp1-mitochondrial fragmentation protects mitochondria. In support, supplementation of good glycemic control, which has followed poor glycemic control, with therapies targeted at regulating mitochondrial dysfunction and biogenesis, is shown to protect the mitochondria from being damaged and prevent the progression of retinopathy and intervention of good glycemia directly with inhibitors of DNA methylation prevent mtDNA damage and prevent the progression of retinopathy [23, 29]. Current evidence strongly favors the expected bioactivity of Mdiv toward Drp1 activity-fission and preclinical models of human diseases have provided promising results, few recent reports suggest that it does not affect mitochondrial morphology in primary neurons, or it acts via inhibiting the complex I of the electron transport chain [47, 51–53], these possibilities cannot be ruled out.

Consistent with the *in vitro* model, retinal microvessels from streptozotocin-induced rats maintained in poor glycemic control for four months, followed by good glycemic control for four additional months, further confirmed the role of Drp1 in the metabolic memory phenomenon associated with continued progression of diabetic retinopathy; diabetes-induced increase in Drp1, its GTPase activity, and in Fis1 remain upregulated even when hyperglycemic insult is halted, suggesting the role of Drp1 in metabolic memory phenomenon associated with continued progression of diabetic retinopathy even after termination of hyperglycemia. The same *in vivo* model has shown suboptimal mitochondrial levels of *Mfn2* and continued development of retinal histopathology and characteristic of diabetic retinopathy, even after removal of hyperglycemic insult [23], suggesting that the retinal vasculature experiences both downregulated fusion process and upregulated fission process, and mitochondria continue to be fragmented, further confirming the role of mitochondrial dynamics in the metabolic memory phenomenon.

## 5. Conclusions

In conclusion, using experimental models, our study demonstrates the role of Drp1 in the metabolic memory associated with the continued progression of diabetic retinopathy. Although the hyperglycemic milieu decreases Mfn2, it also increases Drp1 translocation inside the mitochondria and its GTPase activity. These processes continue to impair mitochondrial dynamics even when the hyperglycemic insult is reversed by normal glycemia. Direct regulation of Drp1 activity, along with normal glucose levels, halts Drp1 activation and maintains mitochondrial dynamics. Thus, by maintaining mitochondrial homeostasis, inhibition of Drp1 activation has the potential to retard the development of diabetic retinopathy and in the metabolic memory associated with its continued progression even when hyperglycemic insult is terminated.

## Figures and Tables

**Table 1:** Human donors.

	Age (years)	Diabetes duration (years)	Cause of death
Nondiabetic donors			
1	70		Myocardial infraction
2	53		Ischemic stroke
3	70		Pulmonary hypertension
4	68		Myocardial infraction
5	73		Myocardial infraction
6	52		Myocardial infraction
7	71		Cerebrovascular accident
8	42		Transient ischemic stroke
Donors with diabetic retinopathy			
1	60	>20	Myocardial infraction
2	73	32	Hypoxia
3	75	35	Gastrointestinal bleed
4	55	16	Intracranial hemorrhage
5	60	25	Myocardial infraction
6	69	22	Subdural hematoma
7	68	16	Myocardial infraction
8	74	38	Myocardial infraction

**Table 2:** Primer sequences.

Primer	Sequence
*Human*	
*Drp1*	Fwd-GAA GGA GGC GAA CTG TGG GC
	Rev-GCA GCT GGA TGA TGT CGG CG
*Fis1*	Fwd-CAG CGG GAT TAC GTC TTC TAC
	Rev-TTC CTT GGC CTG GTT GTT
*Cytb*	Fwd-TCA CCA GAC GCC TCA ACC GC
	Rev-GCC TCG CCC GAT GTG TAG GA
*ACTB*	Fwd-AGC CTC GCC TTT GCC GAT CCG
	Rev-TCT CTT GCT CTG GGC CTC GTCG
*Rat*	
*Drp1*	Fwd-GCA GCC GTA GTC CTC AAA GA
	Rev-CTC CAC CTT TTG AAG CCA GG
*Fis1*	Fwd-ATC TGC TCA CGT CTC CCT CTT T
	Rev-GCT GCC TTC AGG ATT TGG ACT T
*Actb*	Fwd-TCT GTG TGG ATT GGT GGC TCT A
	Rev-AAC AGT CCG CCT AGA AGC ATT TG

## Data Availability

RAK is the guarantor of this work and, as such, has full access to all data in the study and takes responsibility for the integrity of the data and the accuracy of the data analysis.
